# Diminished levels of nasal S100A7 (psoriasin) in seasonal allergic rhinitis: an effect mediated by Th2 cytokines

**DOI:** 10.1186/1465-9921-13-2

**Published:** 2012-01-09

**Authors:** Anne Månsson Kvarnhammar, Camilla Rydberg, Malin Järnkrants, Mia Eriksson, Rolf Uddman, Mikael Benson, Lars-Olaf Cardell

**Affiliations:** 1 Division of ENT Diseases, Department of Clinical Sciences, Intervention and Technology, Karolinska Institutet, Huddinge, Sweden; 2 Laboratory of Clinical and Experimental Allergy Research, Department of Otorhinolaryngology, Skåne University Hospital, Malmö, Sweden; 3 Department of Pediatrics, Linköping University Hospital, Linköping, Sweden

**Keywords:** allergen-specific immunotherapy, antimicrobial peptide, epithelium, lipopolysaccharide, seasonal allergic rhinitis, Th2 cytokines

## Abstract

**Background:**

S100A7 is an antimicrobial peptide involved in several inflammatory diseases. The aim of the present study was to explore the expression and regulation of S100A7 in seasonal allergic rhinitis (SAR).

**Methods:**

Nasal lavage (NAL) fluid was obtained from healthy controls before and after lipopolysaccharide (LPS) provocation, from SAR patients before and after allergen challenge, and from SAR patients having completed allergen-specific immunotherapy (ASIT). Nasal biopsies, nasal epithelial cells and blood were acquired from healthy donors. The airway epithelial cell line FaDu was used for *in vitro *experiments. Real-time RT-PCR and immunohistochemistry were used to determine S100A7 expression in nasal tissue and cells. Release of S100A7 in NAL and culture supernatants was measured by ELISA. The function of recombinant S100A7 was explored in epithelial cells, neutrophils and peripheral blood mononuclear cells (PBMC).

**Results:**

Nasal administration of LPS induced S100A7 release in healthy non-allergic subjects. The level of S100A7 was lower in NAL from SAR patients than from healthy controls, and it was further reduced in the SAR group 6 h post allergen provocation. In contrast, ASIT patients displayed higher levels after completed treatment. S100A7 was expressed in the nasal epithelium and in glands, and it was secreted by cultured epithelial cells. Stimulation with IL-4 and histamine repressed the epithelial S100A7 release. Further, recombinant S100A7 induced activation of neutrophils and PBMC.

**Conclusions:**

The present study shows an epithelial expression and excretion of S100A7 in the nose after microbial stimulation. The levels are diminished in rhinitis patients and in the presence of an allergic cytokine milieu, suggesting that the antimicrobial defense is compromised in patients with SAR.

## Background

S100A7, also known as psoriasin, belongs to the S100 protein family consisting of ~20 different EF-hand type proteins [1]. It was first identified as highly up-regulated in the skin of psoriatic patients [2], but has also been attributed a role in atopic dermatitis (AD) and different types of cancer, including skin, breast and bladder cancer [3-6]. The function of S100A7 is still poorly understood, but several studies suggest that it acts both as an antimicrobial peptide (AMP) and as a chemotactic factor for neutrophils and CD4^+ ^T cells [7-9]. In addition, S100A7 has been shown to directly kill bacteria and protect human skin from *E. coli *infection [7], and to activate neutrophils to produce a range of cytokines, chemokines, AMPs and reactive oxygen species [10].

Although S100A7 has been linked to atopic dermatitis, there is little information available regarding its role in other atopic diseases. We have previously shown lower levels of S100A7 in nasal lavage (NAL) fluid from patients with pollen-induced seasonal allergic rhinitis (SAR) compared to non-allergic controls [11], along with lower S100A7 mRNA levels in tonsils obtained from allergic as compared to healthy donors [12]. Moreover, by analyzing the genetic variation in the *S100A7 *gene, we have described a SNP (rs3014837), which gives rise to a Asp → Glu amino acid shift, that is associated with the occurrence of SAR [13]. In addition, Tieu *et al. *have recently demonstrated diminished levels of S100A7 in NAL fluids from patients with chronic rhinosinusitis (CRS) and SAR, along with a less intense epithelial S100A7 staining in sinonasal tissue extracts from CRS patients compared to control samples [14]. Previous studies suggest the epithelium to be the main cellular source of S100A7 in the nose [14,15], but the mechanisms regulating its differential expression in airway inflammation have not yet been established. Therefore, the purpose of the present study was to further evaluate the expression of S100A7 in the nose and to investigate its regulation in SAR.

## Methods

### Subjects

The study was approved by the local Ethics Committee and all participants gave their written informed consent. A detailed description of the subjects included is presented in Table 1. NAL fluids were obtained from *i) *13 healthy subjects before and 24 h after nasal LPS administration; *ii) *12 patients with SAR before and 6 h after allergen provocation; and *iii) *10 SAR patients prior to and 3 years after completion of allergen-specific immunotherapy (ASIT) with a depot vaccine (Alutard^®^, ALK Abelló, Hørsholm, Denmark). Serum was acquired from 10 healthy volunteers and 12 patients with SAR. Nasal biopsies were taken from 11 healthy volunteers.

**Table 1:** Patient characteristics.

	Treatment	n	Samples	Sex	Age, median (range)
**Healthy subjects**	LPS	13	NAL	5 M, 8 F	27 (22-47)

**SAR patients**	allergen	12	NAL, serum	6 M, 6 F	29 (19-47)

**SAR patients**	ASIT	10	NAL	7M, 3 F	31 (15-36)

**Healthy subjects**	-	10	serum	2 M, 8 F	27.5 (23-48)

**Healthy subjects**	-	11	biopsies	5 M, 6 F	38 (20-50)

**Healthy subjects**	-	6	HNEC	4 M, 2 F	37 (25-48)

ASIT, allergen-specific immunotherapy; F, females; HNEC, human nasal epithelial cells; M, males; NAL, nasal lavage; SAR, seasonal allergic rhinitis

All SAR patients had a history of birch and/or grass pollen-induced rhinoconjunctivitis for at least 2 years with moderate to severe symptoms and exhibited a positive skin prick test (SPT) to birch and/or timothy pollen (wheal reaction diameter > 3 mm) as previously described in detail [16]. Control subjects were all symptom-free with no history of SAR and a negative SPT to the standard panel of allergens. All participants were free from medication ≥ 3 months prior to the study. Subjects with a history or signs of CRS including nasal polyposis were excluded along with patients with hypertrophy of turbinates, severe septum deviation or a history of airway infection within two weeks before the first visit.

### Lipopolysaccharide (LPS) and allergen provocation

Healthy subjects were sprayed with 100 μl of a 0.5 μg/μl solution of LPS from *E. coli *(Sigma-Aldrich, St. Louis, MO, USA) into each nostril, yielding a total of 100 μg LPS [17]. Allergen provocation was performed by spraying 10,000 SQ-U of birch or grass pollen extracts (Aquagen, ALK Abelló) into each nostril. Nasal washings were performed prior to and after provocation.

### Sample collection

All sampling was performed outside pollen season. Blood samples were obtained in Vacuette^® ^serum tubes. NAL fluid was acquired as previously described in detail [18]. Briefly, after clearing excess mucus by exsufflation, a sterile saline solution was aerosolized into both nostrils. Nasal fluids were passively collected using a graded test tube until 7 ml was collected. After centrifugation at 1750 rpm at 4°C for 10 min the supernatant was separated from the cell pellet and collected. Nasal biopsies, approximately 2 × 2 × 2 mm in size, were taken from the inferior turbinate after topical application of local anaesthesia containing lidocaine hydrochloride:nafazoline (34 mg/ml:0.17 mg/ml) for 20 min.

### Airway epithelial cells (AEC)

The nasopharyngeal epithelial cell line FaDu was obtained from ATCC (Manassas, VA, USA) and cultured in Minimum Essential Medium (MEM) with Earle's salts and 2 mM L-glutamine (Gibco, Grand Island, NY, USA) supplemented with 10% FBS (PAN biotech, Aidenbach, Germany), 100 U/ml penicillin and 100 μg/ml streptomycin (Gibco). Primary human nasal epithelial cells (HNEC) were obtained by nasal brushings of 6 healthy non-smoking volunteers (4 males, 2 females, age 20-50) as previously described in detail [19]. HNEC were cultured in collagen-coated tissue culture flasks in airway epithelial cell growth medium supplemented with 0.4% bovine pituitary extract, 10 ng/ml epidermal growth factor, 5 μg/ml insulin, 0.5 μg/ml hydrocortisone, 0.5 μg/ml epinephrine, 6.7 ng/ml triiodothyronine, 10 μg/ml transferrin, 0.1 ng/ml retinoic acid (PromoCell, Heidelberg, Germany), 100 U/ml penicillin and 100 μg/ml streptomycin (Gibco) [20]. All cells were cultured at 37°C in a humidified 5% CO_2 _air atmosphere.

FaDu cells were seeded on 24-well culture plates (250,000 cells/well) in 1 ml complete MEM and incubated overnight. Thereafter, they were cultured for additionally 24 h in the absence or presence of recombinant human IL-4 (R&D Systems, Minneapolis, MN, USA), histamine (Sigma-Aldrich) or S100A7 (ProtEra, Sesto Fiorentino, Italy).

### Isolation and culture of cells from peripheral blood

Peripheral blood was acquired from healthy volunteers, diluted 1:1 in PBS and centrifuged using Ficoll-Paque (Amersham Bioscience, Uppsala, Sweden). Peripheral blood mononuclear cells (PBMC) were retrieved from the interphase fraction, whereas granulocytes were recovered from the pellet. Briefly, the granulocyte-rich pellet, containing ≥ 90% neutrophils, was treated with an ammonium chloride lysis buffer to remove erythrocytes, followed by collection of neutrophils. Eosinophils were negatively selected from granulocytes using the MACS magnetic labeling system (Eosinophil Isolation Kit, Miltenyi Biotec, Cologne, Germany) as previously described in detail [21]. The interphase fraction after centrifugation was washed, and monocytes were isolated using CD14 Microbeads (Miltenyi Biotec). B and T lymphocytes were negatively selected using the B cell Isolation Kit II and Pan T cell Isolation Kit II [22,23], respectively (Miltenyi Biotec).

PBMC (1 × 10^6 ^cells/ml) and neutrophils (4 × 10^6 ^cells/ml) were cultured in RPMI 1640 supplemented with 0.3 g/l L-glutamine (PAA, Pasching, Austria), 10% FBS, 100 U/ml streptomycin and 100 μg/ml penicillin in the absence or presence of rhS100A7. After various time points, cytokine levels in the supernatants were analyzed using ELISA, proliferation was measured by [*methyl*-^3^H]thymidine incorporation and expression of the adhesion molecule CD11b was determined using flow cytometry.

### Immunohistochemistry

The presence of S100A7 protein in the nose was assessed using immunohistochemical staining. Nasal biopsies were fixed in 4% buffered formaldehyde for 7 days, thereafter processed and embedded in paraffin. Sections (3 μm thick) were cut, dried for 2 h at 60°C and then stored at 4°C until further use. The tissue sections were deparaffinized in xylene and then rehydrated through graded alcohol and deionized water. For antigen retrieval the sections were processed in a microwave oven in target retrieval solution with a pH of 6.1 (Dako, Glostrup, Denmark). A mouse anti-human S100A7 antibody (clone 47C1068) from Abcam (Cambridge, UK) was used at a dilution of 1:100. As negative control, N-series Universal Negative Control Reagent against mouse (Dako) was utilized. The staining procedure was performed in a Techmate 500 Plus immunostaining machine according to standard "ENVP" procedure (Dako). The sections were counterstained with hematoxylin, dehydrated in graded alcohol, passed through xylene and mounted.

### RNA extraction and real-time RT-PCR

Nasal biopsies, epithelial cells and freshly isolated leukocytes were lysed in RLT buffer (Qiagen, Hilden, Germany) supplemented with 1% 2-mercaptoethanol and stored in -80°C until use. Total RNA was extracted using RNeasy Mini Kit (Qiagen), and the quantity and quality of the RNA were measured by spectrophotometry using the wavelength absorption ratio (260/280 nm). Reverse transcription of total RNA into cDNA was performed using the Omniscript™ reverse transcriptase kit (Qiagen) with oligo(dT)16 primer (DNA Technology A/S, Aarhus, Denmark) in a Mastercycler personal PCR machine (Eppendorf AG, Hamburg, Germany). The reaction was carried out at 37°C for 1 h.

The real-time PCR reactions were performed on a Stratagene Mx3000P (Agilent Technologies, Santa Clara, CA, USA) using the Brilliant^® ^II SYBR^® ^Green QPCR Mastermix (Agilent Technologies) in a final volume of 20 μl. The PCR primers (β-actin fwd: 5'-GCCAACCGCGAGAAGATG-3', rev: 5'ACGGCCAGAGGCGTACAG-3'; S100A7 fwd: CGTGACGCTTCCCAGCTC-3', rev: 5'-TCATCACGTCTGGTGTATTTGTGA-3') were designed using Primer Express^® ^2.0 Software (Applied Biosystems, Foster City, CA, USA) and synthesized by DNA Technology [12]. The thermal cycler was set to perform 95°C for 15 min, followed by 46 cycles of 94°C for 30 s and 55°C for 60 s (initially 65°C, followed by a 2°C decrease for the six first cycles). Melting curve analysis was performed to ensure specificity of the amplified PCR products. The mRNA expression was assessed using the comparative cycle threshold (Ct) method where the relative amounts of mRNA were determined by subtracting the Ct value of S100A7 with Ct values of β-actin (ΔCt). The amount of mRNA is expressed as 2^-ΔCt ^× 10^5 ^[19-23].

### Flow cytometry

Cultured neutrophils were stained with CD16-ECD (clone 3G8) and CD11b-FITC (Bear1) (Beckman Coulter, Marseille, France) for 15 min in RT. Thereafter, cells were washed, resuspended in PBS and analyzed on a Coulter Epics XL flow cytometer (Beckman Coulter). Neutrophils were distinguished based on their forward scatter (FSc) and side scatter (SSc) properties as well as their expression of CD16, as FSc^high^, SSc^high^, CD16^+^. 10,000-15,000 events were collected and analyzed using Expo32 ADC analysis software (Beckman Coulter).

### ELISA

Levels of S100A7 in unprocessed NAL fluid, serum and culture supernatants were detected using the Circulex™ S100A7/Psoriasin ELISA kit (detection limit 0.12 ng/ml) from MBL International (Woburn, MA, USA). ELISA plates to measure IL-1β (1 pg/ml), IL-6 (0.7 pg/ml), IL-8 (3.5 pg/ml) and IL-10 (3.9 pg/ml) in cell culture supernatants were obtained from R&D Systems.

### Statistics

Statistical analyses were performed using GraphPad Prism 5 (San Diego, CA, USA). All data are presented as mean ± standard error of the mean (SEM), and *n *equals the number of independent donors. For unpaired data, statistical differences were determined using unpaired Student's *t*-test with Welch correction if the variances were non-homogenous. Paired observations were analyzed using paired *t*-test (for two sets of data) or one-way repeated measures ANOVA with Dunnett's post test (> 2 sets of data). A *p*-value of < 0.05 was considered statistically significant.

## Results

### Nasal LPS administration induces release of S100A7

**Figure 1:**
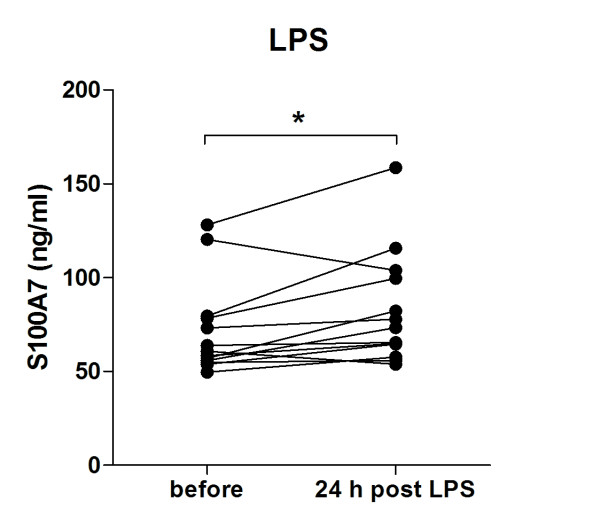
**Nasal administration of LPS induces S100A7 release**. Healthy subjects were exposed to nasal administration of LPS (100 μg). Nasal lavage (NAL) fluids were obtained before and 24 h post LPS challenge, followed by measurements of S100A7 by use of ELISA (n = 13). *, p < 0.05 (paired *t*-test).

To investigate whether microbial exposure affects the nasal release of S100A7, healthy non-allergic subjects were exposed to nasal administration of the prototypical inflammatory stimulus LPS. NAL fluids were collected before and 24 h post challenge followed by measurements of S100A7 by ELISA. LPS was found to increase the S100A7 excretion (Figure 1).

### Levels of S100A7 in NAL fluid are lower in patients with SAR and decrease after allergen provocation

**Figure 2:**
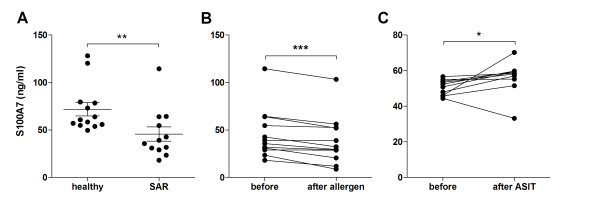
**Diminished levels of nasal S100A7 in allergic patients**. **(A) **Baseline levels of S100A7 were measured by ELISA in nasal lavage (NAL) fluids obtained from healthy subjects (n = 13) and patients with seasonal allergic rhinitis (SAR, n = 12). **, p < 0.01 (unpaired *t*-test). **(B) **Levels of S100A7 in NAL fluid from patients with SAR before and 6 h after allergen provocation (n = 12). ***, p < 0.001 (paired *t*-test). (C) Baseline levels of S100A7 in NAL fluid obtained from patients with SAR before and 3 years after allergen-specific immunotherapy (ASIT, n = 10). *, p < 0.05 (paired t-test).

NAL fluids and serum from healthy subjects and SAR patients outside season were analyzed for levels of S100A7 by use of ELISA. A significantly lower baseline S100A7 release in the nose was seen in the allergic as compared to the healthy group (Figure 2A), whereas no difference was found in serum (4.5 ± 0.9 *vs*. 3.7 ± 0.5 ng/ml; p = 0.74). Thereafter, we wanted to investigate the effects of allergen provocation on nasal S100A7 secretion in the allergic group. It was found that the S100A7 levels were significantly lower 6 h post provocation as compared to before (Figure 2B).

### Levels of S100A7 increase in SAR patients after ASIT

The diminished levels among allergics led us to investigate whether SAR patients that have completed ASIT treatment still have a deficient S100A7 production or whether they have acquired levels comparable to those of healthy individuals. NAL fluid was collected from 10 patients before and after ASIT and analyzed for baseline levels of S100A7 by use of ELISA. Immunotherapy treatment increased the generation of S100A7 in nine out of the ten donors examined (Figure 2C).

### S100A7 is produced by nasal epithelial cells

**Figure 3:**
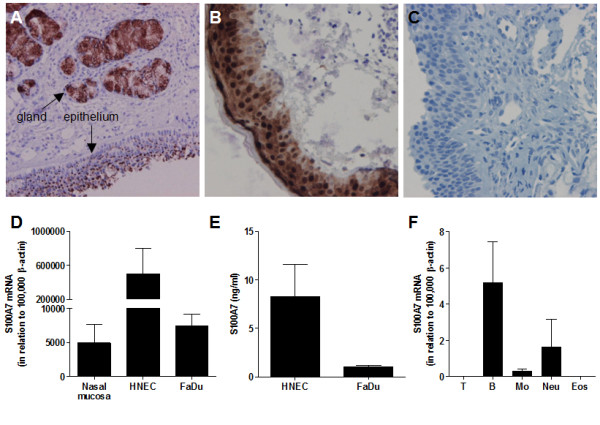
**S100A7 is present in the nasal epithelium and seromucous glands**. Tissue slides of the human inferior turbinate were stained with **(A, B) **a monoclonal antibody against S100A7 or **(C) **an isotype-matched control antibody. Positive staining (brown) was detected in the nasal epithelium and in seromucous glands. Counterstaining with hematoxylin (blue) (magnification 100 ×). **(D) **Expression of S100A7 mRNA was determined in nasal biopsies (n = 11), primary human nasal epithelial cells (HNEC, n = 6) and FaDu (n = 4) by real-time RT-PCR. Data is expressed in relation to β-actin as 2^-ΔCt ^× 10^5^. **(E) **Levels of S100A7 in the culture medium from HNEC and FaDu were analyzed by ELISA (n = 4-6). **(F) **mRNA expression of S100A7 in purified peripheral blood T lymphocytes, B lymphocytes, monocytes, neutrophils and eosinophils using real-time RT-PCR (n = 3-6). Data are presented as mean ± SEM.

To ascertain the presence of S100A7 in the nose, immunohistochemistry and real-time RT-PCR were performed on nasal biopsies obtained from healthy donors. An intense immunostaining was seen in seromucous glands and in the epithelium (Figure 3A, B), whereas replacement of the primary specific antibody with an isotype-matched control antibody resulted in complete loss of staining (Figure 3C). Expression of S100A7 mRNA was found in all biopsy specimens (Figure 3D).

The cellular sources of S100A7 were further investigated in primary HNEC, the nasopharyngeal epithelial cell line FaDu and isolated leukocytes that can be found in the epithelial and subepithelial regions. A distinct mRNA expression was seen in HNEC, but also in FaDu (Figure 3D), and secreted S100A7 could be detected in the cell culture medium from both AECs (Figure 3E). Low levels of S100A7 mRNA were also found in B cells, monocytes and neutrophils, whereas there was no expression in T cells or eosinophils (Figure 3F).

### Th2 cytokines repress the S100A7 secretion by epithelial cells

To study the regulation of S100A7 in the nose and elucidate the mechanisms behind the diminished levels during SAR, we hypothesized that it might be consumed during the allergic inflammation by the infiltrating cells. The target cells for secreted S100A7 in the nose are not fully identified, but the reported chemotactic properties for CD4^+ ^T cells and neutrophils [8] suggest that it is aimed for the latter as neutrophils are the most abundant cell type in nasal washings [24]. The number of neutrophils in NAL fluid was significantly higher in the allergic as compared to the healthy group (6.4 ± 1.8 *vs*. 1.6 ± 0.7 × 10^4 ^cells/ml; p < 0.05), whereas allergen provocation did not alter the number of cells (5.7 ± 0.8 *vs*. 6.4 ± 1.8 × 10^4 ^cells/ml; p = 0.70). However, no correlation between neutrophil numbers and S100A7 levels was found (r^2^= 0.055; Pearson r = -0.235; p = 0.21).

**Figure 4:**
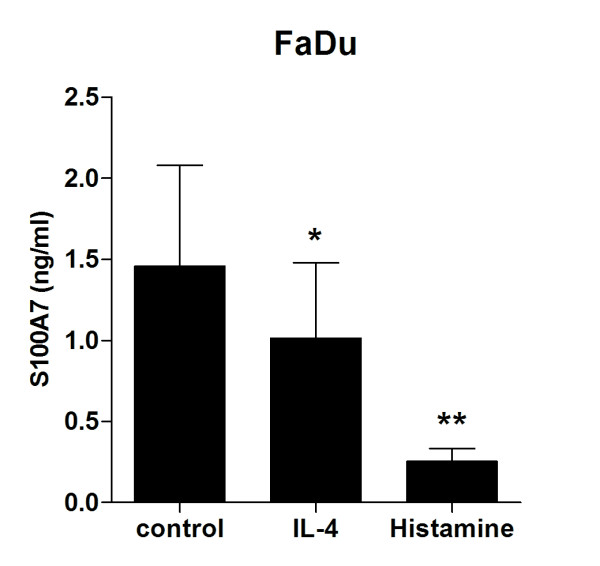
**IL-4 and histamine repress the S100A7 secretion by epithelial cells**. The nasopharyngeal epithelial cell line FaDu was cultured in the absence or presence of IL-4 (10 ng/ml) or histamine (10 μM) for 24 h followed by measurements of S100A7 in the culture supernatants by use of ELISA (n = 6). Data are presented as mean ± SEM. *, p < 0.05 (paired *t*-test).

Instead, we asked whether mediators produced during the allergic inflammation *per se *could have a role in the regulation of S100A7. FaDu was cultured for 24 h in the absence or presence of IL-4 and histamine followed by measurements of S100A7 levels. Both mediators markedly repressed the epithelial S100A7 release (Figure 4).

### S100A7 activates PBMC and neutrophils

**Figure 5:**
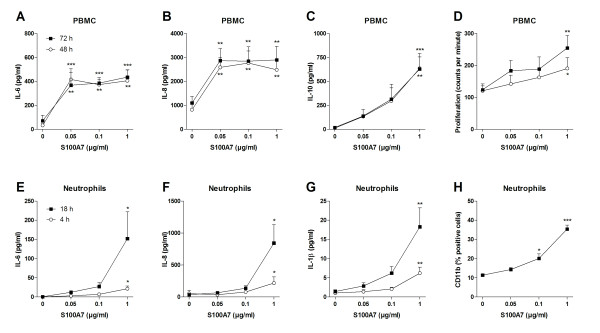
**S100A7 activates PBMC and neutrophils**. **(A-D) **PBMC were cultured (1 × 10^6 ^cells/ml) for 48 and 72 h with or without S100A7. Thereafter, cell culture supernatants were analyzed for levels of IL-6, IL-8 and IL-10 by use of ELISA, and the proliferative capacity of the cells was determined by [*methyl*-^3^H]thymidine incorporation (n = 6). **(E-H) **Neutrophils were cultured (4 × 10^6 ^cells/ml) for 4 and 18 h in the absence or presence of S100A7. Levels of IL-6, IL-8 and IL-1β were measured in the supernatants by ELISA, and expression of CD11b was analyzed by flow cytometry (n = 6). Data are expressed as mean ± SEM. *, p < 0.05; **, p < 0.01; ***, p < 0.001 (ANOVA).

The last set of experiments aimed to explore the ability of S100A7 to activate different cellular subsets. The epithelial cell line FaDu, PBMC and neutrophils were stimulated with rhS100A7 for various time points followed by measurements of cytokine secretion, proliferation and adhesion molecule expression. In epithelial cells, S100A7 promoted neither IL-6 nor IL-8 secretion after 24 h of stimulation (data not shown). PBMC, on the other hand, were induced to release IL-6, IL-8 and IL-10, as well as proliferate in response to S100A7 (Figure 5A-D). Likewise, neutrophils were activated to produce IL-1β, IL-6 and IL-8, and up-regulate the expression of the adhesion molecule CD11b (Figure 5E-H).

## Discussion

The present study describes S100A7 in the nose and how it is regulated during SAR. Nasal administration of LPS induces S100A7 release in healthy non-allergic subjects. Lower levels of S100A7 are seen in NAL fluid from SAR patients than from healthy controls and the levels are further decreased upon allergen provocation. SAR patients having completed ASIT display higher levels of S100A7 in NAL than before initiation of treatment. S100A7 is found to emanate mainly from the nasal epithelium and to activate PBMC and neutrophils to produce cytokines, proliferate and up-regulate adhesion molecule expression. Further, the epithelium-induced S100A7 generation is repressed by the allergic mediators IL-4 and histamine.

We have previously identified S100A7 in the NAL proteome using 2-dimensional gel electrophoresis in combination with mass spectrometry, and demonstrated lower levels in patients with symptomatic SAR compared to controls [11]. The present study, investigating S100A7 in NAL using ELISA, confirms and complements previous data by showing diminished levels in SAR patients outside pollen season compared to non-allergic controls, along with a further reduction in the SAR group after allergen provocation. Moreover, the levels appear to return to normal after immunotherapy treatment. No differences in S100A7 levels among healthy and allergic subjects are seen in serum, which supports the notion of a local role for S100A7 in SAR. Results from immunohistochemical staining and real-time RT-PCR of nasal biopsies clearly demonstrate presence of S100A7 in the epithelium and seromucous glands, and culture of epithelial cells reveals a basal secretion of S100A7. These findings are in accordance with a study by Glaser *et al. *showing an expression in sebaceous glands in the nose [7], and recent results by Tieu and colleagues demonstrating reduced levels of epithelial S100A7 in patients with CRS and SAR [14,15]. It should also be mentioned that in contrast to the low levels of S100A7 seen during inflammatory conditions in the upper airways, there are studies demonstrating an enhanced expression and secretion of S100A7 in the skin of patients with AD [25-27]. Moreover, we have previously detected S100A7 in tonsillar epithelium and lymphocytes (CD19^+^, CD4^+ ^and CD8^+ ^cells) and found reduced levels in tonsils in response to infection and atopic predisposition [12]. In contrast, blood-derived B and T cells express very low or no levels of S100A7. These discrepancies in expression might be related to compartmental differences. Also, the high antigen load in the tonsils might cause a microbe-induced regulation of S100A7 expression.

Accumulating evidence suggests that there might be intrinsic or disease-driven deficiencies in the epithelial barrier function of the nasal mucosa in AR patients [28]. Indeed, allergics suffer from an ongoing so called minimal persistent inflammation, characterized by e.g. infiltration of inflammatory cells (eosinophils and neutrophils) and up-regulation of epithelial adhesion molecules, which in turn primes the nasal mucosa leading to increased sensitivity and responsiveness to allergen provocation [29,30]. Likewise, we have recently shown that SAR patients have higher clinical symptom scores, pulmonary nitric oxide production, NAL leukocyte numbers and cytokine levels compared to healthy subjects [16]. The epithelium provides not only a physical barrier through mucociliary clearance and tight junctions, but it can also resist entry of pathogens through the production of antimicrobial defense proteins. The present study shows reduced levels of S100A7 in SAR patients and in response to allergen challenge. In addition to the epithelial disruption seen in the atopic airway [31], we propose defects in AMPs of this kind to be a factor behind the increased susceptibility to microbial infection often seen during periods of SAR [32-34]. However, patients having completed ASIT have a less pronounced minimal persistent inflammation, manifested by a reduced eosinophilic infiltration into the nasal mucosa and reduced epithelial damage [35], which in turn can explain the higher levels of S100A7 in NAL fluid among these patients.

Nasal challenge with LPS, as a mimic of bacterial upper airway infection, induces release of S100A7 in NAL fluid from healthy subjects. In line with this, the expression of S100A7 by the human hair follicle epithelium has been found to be inducible upon treatment with prototypical microbial products such as LPS [36]. Interestingly, unpublished data from our lab show that nasal administration of LPS (total dose of 50 μg) in SAR patients does not induce release of S100A7 in NAL (before: 92.2 ± 6.5 *vs*. 24 h after: 92.0 ± 7.4, p = 0.98). The inability of these patients to respond in terms of S100A7 secretion might be a consequence of their impaired antimicrobial defense system. However, it cannot be excluded that the differential responsiveness to LPS among healthy and allergic subjects is related to the dose, as a 2-fold lower concentration was given to the latter group.

The diminished levels of S100A7 during SAR do not correlate with the infiltration of neutrophils. Instead, IL-4 and histamine are found to suppress the S100A7 secretion by epithelial cells, suggesting that a Th2-like cytokine milieu negatively regulates the S100A7 production. Elevated levels of Th2-biased cytokines have been demonstrated in nasal washings of SAR patients both during pollen season and after allergen provocation [37-39], and Glaser *et al. *have demonstrated that the TNF-α-induced S100A7 secretion by human keratinocytes is inhibited by IL-4 and IL-13 [25].

Even though there is a ~2-fold reduction in the concentration of S100A7 in NAL fluid from SAR patients, it should be mentioned that the changes in levels in some of the treatment groups are fairly small, which raises questions regarding the biological significance. However, in real-life, efficient clearance of microbes involves a crosstalk between several different cells, signals and mediators that are triggered simultaneously. This is reflected by e.g. the ability of S100A7 to activate the cellular immunity and the ability of cytokines, including TNF-α, IL-4, IL-6 and oncostatin-M [3,25], to both positively and negatively regulate the generation of S100A7. Hence, it is not the effect of each single AMP, but the combined actions of different mediators that are of biological importance.

The receptor for secreted S100A7 is still unknown, and consequently its functional relevance is poorly understood. In the present study, we demonstrate that S100A7 activates human PBMC and neutrophils to produce cytokines, proliferate and up-regulate adhesion molecule expression. Neutrophils have previously been reported to secrete cytokines, reactive oxygen species and AMPs in response to S100A7 stimulation [10], but so far there are no other studies demonstrating an effect on mononuclear cells except for its chemotactic properties for CD4^+ ^T cells [8].

## Conclusion

**Figure 6:**
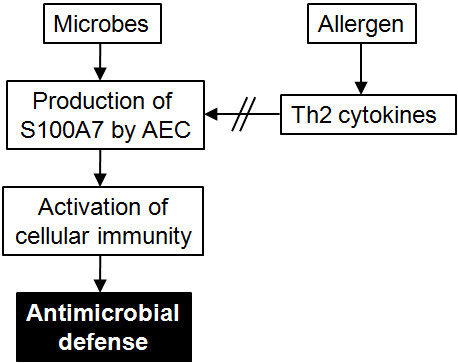
**Expression and regulation of S100A7 in allergic rhinitis**. S100A7 is produced by airway epithelial cells (AEC) upon microbial exposure and triggers activation of immune cells, including neutrophils and mononuclear cells. The bactericidal properties of S100A7 and the activation of the cellular immunity mount an effective antimicrobial response. During seasonal allergic rhinitis, the Th2-driven immune response represses the production of epithelial S100A7, hence increasing the likelihood of microbial colonization or infection.

Present data propose that the nasal epithelium produces S100A7 in response to microbial stimulation, which in turn attracts immunocompetent cells in order to mount a proper antimicrobial defense (Figure 6). However, during an allergic inflammation, the Th2-type cytokine response represses the S100A7 release. As a result of the diminished S100A7 levels, allergic patients have reduced bactericidal properties as well as a decreased capability to activate the cellular immunity, which in turn might render them more susceptible to microbial colonization and/or make their conditions worse once an infection is established. In line with this, it is tempting to speculate that ASIT-treated patients, who have regained a normal S100A7 production, in turn have a reduced propensity to develop upper respiratory tract infections compared to their untreated counterparts.

## List of abbreviations

AEC: airway epithelial cells; AD: atopic dermatitis; AMP: antimicrobial peptide; ASIT: allergen-specific immunotherapy; CRS: chronic rhinosinusitis; Ct: cycle threshold; HNEC: human nasal epithelial cells; LPS: lipopolysaccharide; NAL: nasal lavage; PBMC: peripheral blood mononuclear cells; rh: recombinant human; SAR: seasonal allergic rhinitis; SPT: skin prick test

## Competing interests

The authors declare that they have no competing interests.

## Authors' contributions

AMK performed most of the experiments, analyzed the data and wrote the paper. CR carried out the experiments with the primary epithelial cells. MJ performed the nasal biopsy experiments, ME the neutrophil and PBMC cultures and RU the immunohistochemical analyses. MB helped with the design of the study and revised the manuscript. LOC provided financial support and critically revised the manuscript. All authors have read and approved the final form of the manuscript.

## References

[B1] Marenholz I Heizmann CW Fritz G S100 proteins in mouse and man: from evolution to function and pathology (including an update of the nomenclature) Biochem Biophys Res Commun 2004 322 4 1111 1122 10.1016/j.bbrc.2004.07.096 15336958

[B2] Madsen P Rasmussen HH Leffers H Honore B Dejgaard K Olsen E Kiil J Walbum E Andersen AH Basse B Molecular cloning, occurrence, and expression of a novel partially secreted protein "psoriasin" that is highly up-regulated in psoriatic skin J Invest Dermatol 1991 97 4 701 712 10.1111/1523-1747.ep12484041 1940442

[B3] West NR Watson PH S100A7 (psoriasin) is induced by the proinflammatory cytokines oncostatin-M and interleukin-6 in human breast cancer Oncogene 2010 29 14 2083 2092 10.1038/onc.2009.488 20101226

[B4] Zhang H Zhao Q Chen Y Wang Y Gao S Mao Y Li M Peng A He D Xiao X Selective expression of S100A7 in lung squamous cell carcinomas and large cell carcinomas but not in adenocarcinomas and small cell carcinomas Thorax 2008 63 4 352 359 10.1136/thx.2007.087015 18364444

[B5] Yao R Lopez-Beltran A Maclennan GT Montironi R Eble JN Cheng L Expression of S100 protein family members in the pathogenesis of bladder tumors Anticancer Res 2007 27 5A 3051 3058 17970044

[B6] Moubayed N Weichenthal M Harder J Wandel E Sticherling M Glaser R Psoriasin (S100A7) is significantly up-regulated in human epithelial skin tumours J Cancer Res Clin Oncol 2007 133 4 253 261 10.1007/s00432-006-0164-y 17136347 PMC12160872

[B7] Glaser R Harder J Lange H Bartels J Christophers E Schroder JM Antimicrobial psoriasin (S100A7) protects human skin from Escherichia coli infection Nat Immunol 2005 6 1 57 64 10.1038/ni1142 15568027

[B8] Jinquan T Vorum H Larsen CG Madsen P Rasmussen HH Gesser B Etzerodt M Honore B Celis JE Thestrup-Pedersen K Psoriasin: a novel chemotactic protein J Invest Dermatol 1996 107 1 5 10 10.1111/1523-1747.ep12294284 8752830

[B9] Wolf R Howard OM Dong HF Voscopoulos C Boeshans K Winston J Divi R Gunsior M Goldsmith P Ahvazi B Chemotactic activity of S100A7 (Psoriasin) is mediated by the receptor for advanced glycation end products and potentiates inflammation with highly homologous but functionally distinct S100A15 J Immunol 2008 181 2 1499 1506 18606705 10.4049/jimmunol.181.2.1499 PMC2435511

[B10] Zheng Y Niyonsaba F Ushio H Ikeda S Nagaoka I Okumura K Ogawa H Microbicidal protein psoriasin is a multifunctional modulator of neutrophil activation Immunology 2008 124 3 357 367 10.1111/j.1365-2567.2007.02782.x 18194266 PMC2440830

[B11] Bryborn M Adner M Cardell LO Psoriasin, one of several new proteins identified in nasal lavage fluid from allergic and non-allergic individuals using 2-dimensional gel electrophoresis and mass spectrometry Respir Res 2005 6 118 10.1186/1465-9921-6-118 16236163 PMC1282590

[B12] Bryborn M Mansson A Cardell LO Adner M Differentiated S100A7 expression in infected tonsils and tonsils from allergic individuals FEMS Immunol Med Microbiol 2008 53 3 413 420 10.1111/j.1574-695X.2008.00444.x 18625016

[B13] Bryborn M Hallden C Sall T Adner M Cardell LO Comprehensive evaluation of genetic variation in S100A7 suggests an association with the occurrence of allergic rhinitis Respir Res 2008 9 29 10.1186/1465-9921-9-29 18373864 PMC2335106

[B14] Tieu DD Peters AT Carter RT Suh L Conley DB Chandra R Norton J Grammer LC Harris KE Kato A Evidence for diminished levels of epithelial psoriasin and calprotectin in chronic rhinosinusitis J Allergy Clin Immunol 2010 125 3 667 675 10.1016/j.jaci.2009.11.045 20226301 PMC2841036

[B15] Richer SL Truong-Tran AQ Conley DB Carter R Vermylen D Grammer LC Peters AT Chandra RK Harris KE Kern RC Epithelial genes in chronic rhinosinusitis with and without nasal polyps Am J Rhinol 2008 22 3 228 234 10.2500/ajr.2008.22.3162 18588753 PMC2810157

[B16] Mansson A Bachar O Adner M Cardell LO Nasal CpG oligodeoxynucleotide administration induces a local inflammatory response in nonallergic individuals Allergy 2009 64 9 1292 1300 10.1111/j.1398-9995.2009.02012.x 19243360

[B17] Ekman AK Fransson M Rydberg C Adner M Cardell LO Nasal Challenge with LPS Stimulates the Release of Macrophage Inflammatory Protein 1alpha Int Arch Allergy Immunol 2009 149 2 154 160 10.1159/000189199 19127073

[B18] Benson M Strannegard IL Wennergren G Strannegard O Interleukin-5 and interleukin-8 in relation to eosinophils and neutrophils in nasal fluids from school children with seasonal allergic rhinitis Pediatr Allergy Immunol 1999 10 3 178 185 10565558 10.1034/j.1399-3038.1999.00036.x

[B19] Rydberg C Mansson A Uddman R Riesbeck K Cardell LO Toll-like receptor agonists induce inflammation and cell death in a model of head and neck squamous cell carcinomas Immunology 2009 128 1 Suppl e600 611 19740321 10.1111/j.1365-2567.2008.03041.x PMC2753959

[B20] Bogefors J Rydberg C Uddman R Fransson M Mansson A Benson M Adner M Cardell LO Nod1, Nod2 and Nalp3 receptors, new potential targets in treatment of allergic rhinitis? Allergy 2010 65 10 1222 1226 10.1111/j.1398-9995.2009.02315.x 20384614

[B21] Mansson A Cardell LO Role of atopic status in Toll-like receptor (TLR)7- and TLR9-mediated activation of human eosinophils J Leukoc Biol 2009 85 4 719 727 10.1189/jlb.0808494 19129482

[B22] Mansson A Adner M Hockerfelt U Cardell LO A distinct Toll-like receptor repertoire in human tonsillar B cells, directly activated by PamCSK, R-837 and CpG-2006 stimulation Immunology 2006 118 4 539 548 16780564 10.1111/j.1365-2567.2006.02392.x PMC1782320

[B23] Mansson A Adner M Cardell LO Toll-like receptors in cellular subsets of human tonsil T cells: altered expression during recurrent tonsillitis Respir Res 2006 7 36 10.1186/1465-9921-7-36 16504163 PMC1481585

[B24] Kinhult J Adner M Uddman R Cardell LO Pituitary adenylate cyclase-activating polypeptide, effects in the human nose Clin Exp Allergy 2003 33 7 942 949 10.1046/j.1365-2222.2003.01721.x 12859451

[B25] Glaser R Meyer-Hoffert U Harder J Cordes J Wittersheim M Kobliakova J Folster-Holst R Proksch E Schroder JM Schwarz T The antimicrobial protein psoriasin (S100A7) is upregulated in atopic dermatitis and after experimental skin barrier disruption J Invest Dermatol 2009 129 3 641 649 10.1038/jid.2008.268 18754038

[B26] Sugiura H Ebise H Tazawa T Tanaka K Sugiura Y Uehara M Kikuchi K Kimura T Large-scale DNA microarray analysis of atopic skin lesions shows overexpression of an epidermal differentiation gene cluster in the alternative pathway and lack of protective gene expression in the cornified envelope Br J Dermatol 2005 152 1 146 149 10.1111/j.1365-2133.2005.06352.x 15656815

[B27] Harder J Dressel S Wittersheim M Cordes J Meyer-Hoffert U Mrowietz U Folster-Holst R Proksch E Schroder JM Schwarz T Enhanced expression and secretion of antimicrobial peptides in atopic dermatitis and after superficial skin injury J Invest Dermatol 2010 130 5 1355 1364 10.1038/jid.2009.432 20107483

[B28] Tieu DD Kern RC Schleimer RP Alterations in epithelial barrier function and host defense responses in chronic rhinosinusitis J Allergy Clin Immunol 2009 124 1 37 42 10.1016/j.jaci.2009.04.045 19560577 PMC2802265

[B29] Canonica GW Compalati E Minimal persistent inflammation in allergic rhinitis: implications for current treatment strategies Clin Exp Immunol 2009 158 3 260 271 10.1111/j.1365-2249.2009.04017.x 19765020 PMC2792821

[B30] Ricca V Landi M Ferrero P Bairo A Tazzer C Canonica GW Ciprandi G Minimal persistent inflammation is also present in patients with seasonal allergic rhinitis J Allergy Clin Immunol 2000 105 1 Pt 1 54 57 10629452 10.1016/s0091-6749(00)90177-5

[B31] Langley SJ Goldthorpe S Craven M Woodcock A Custovic A Relationship between exposure to domestic allergens and bronchial hyperresponsiveness in non-sensitised, atopic asthmatic subjects Thorax 2005 60 1 17 21 10.1136/thx.2004.027839 15618577 PMC1747172

[B32] Micillo E Bianco A D'Auria D Mazzarella G Abbate GF Respiratory infections and asthma Allergy 2000 55 Suppl 61 42 45 10.1034/j.1398-9995.2000.00506.x 10919505

[B33] Sykes A Johnston SL Etiology of asthma exacerbations J Allergy Clin Immunol 2008 122 4 685 688 10.1016/j.jaci.2008.08.017 19014758

[B34] Newcomb DC Peebles RS Jr Bugs and asthma: a different disease? Proc Am Thorac Soc 2009 6 3 266 271 10.1513/pats.200806-056RM 19387028 PMC2677401

[B35] Lauriello M Muzi P Di Rienzo L Di Stanislao C Tirelli GC Bologna M A two-year course of specific immunotherapy or of continuous antihistamine treatment reverse eosinophilic inflammation in severe persistent allergic rhinitis Acta Otorhinolaryngol Ital 2005 25 5 284 291 16602327 PMC2639903

[B36] Reithmayer K Meyer KC Kleditzsch P Tiede S Uppalapati SK Glaser R Harder J Schroder JM Paus R Human hair follicle epithelium has an antimicrobial defence system that includes the inducible antimicrobial peptide psoriasin (S100A7) and RNase 7 Br J Dermatol 2009 161 1 78 89 10.1111/j.1365-2133.2009.09154.x 19416233

[B37] Benson M Strannegard IL Wennergren G Strannegard O Cytokines in nasal fluids from school children with seasonal allergic rhinitis Pediatr Allergy Immunol 1997 8 3 143 149 10.1111/j.1399-3038.1997.tb00168.x 9532255

[B38] Erin EM Zacharasiewicz AS Nicholson GC Tan AJ Higgins LA Williams TJ Murdoch RD Durham SR Barnes PJ Hansel TT Topical corticosteroid inhibits interleukin-4, -5 and -13 in nasal secretions following allergen challenge Clin Exp Allergy 2005 35 12 1608 1614 10.1111/j.1365-2222.2005.02381.x 16393327

[B39] Kosa L Kovacs N Halasz A Interleukines in nasal lavage Allergy 2002 57 1 63 64 11991302

